# Lifestyle factors and risk of sickness absence from work: a multicohort study

**DOI:** 10.1016/S2468-2667(18)30201-9

**Published:** 2018-11-05

**Authors:** Marianna Virtanen, Jenni Ervasti, Jenny Head, Tuula Oksanen, Paula Salo, Jaana Pentti, Anne Kouvonen, Ari Väänänen, Sakari Suominen, Markku Koskenvuo, Jussi Vahtera, Marko Elovainio, Marie Zins, Marcel Goldberg, Mika Kivimäki

**Affiliations:** a Department of Public Health and Caring Sciences, University of Uppsala, Uppsala, Sweden; b Finnish Institute of Occupational Health, Helsinki and Turku, Finland; c Department of Epidemiology and Public Health, University College London, London, UK; d Department of Psychology, University of Turku, Turku, Finland; e Department of Public Health, University of Turku and Turku University Hospital, Turku, Finland; f Faculty of Social Sciences, University of Helsinki, Helsinki, Finland; g Department of Psychology and Logopedics, University of Helsinki, Helsinki, Finland; h SWPS University of Social Sciences and Humanities, Wroclaw, Poland; i School of Social Policy, Sociology and Social Research, University of Kent, UK; j University of Skövde, Skövde, Sweden; k Folkhälsan Research Center, Helsinki, Finland; l Clinicum, Faculty of Medicine, University of Helsinki, Finland; m National Institute for Health and Welfare, Helsinki, Finland; n Inserm, Population-based Epidemiologic Cohorts Unit UMS 011, Villejuif, France; o Paris Descartes University, Paris, France

## Abstract

**Background:**

Lifestyle factors influence the risk of morbidity and mortality, but the extent to which they are associated with employees' absence from work due to illness is unclear. We examined the relative contributions of smoking, alcohol consumption, high body-mass index, and low physical activity to diagnosis-specific sickness absence.

**Methods:**

We did a multicohort study with individual-level data of participants of four cohorts from the UK, France, and Finland. Participants' responses to a lifestyle survey were linked to records of sickness absence episodes, typically lasting longer than 9 days; for each diagnostic category, the outcome was the total number of sickness absence days per year. We estimated the associations between lifestyle factors and sickness absence by calculating rate ratios for the number of sickness absence days per year and combining cohort-specific estimates with meta-analysis. The criteria for assessing the evidence included the strength of association, consistency across cohorts, robustness to adjustments and multiple testing, and impact assessment by use of population attributable fractions (PAF), with both internal lifestyle factor prevalence estimates and those obtained from European populations (PAF_external_).

**Findings:**

For 74 296 participants, during 446 478 person-years at risk, the most common diagnoses for sickness absence were musculoskeletal diseases (70·9 days per 10 person-years), depressive disorders (26·5 days per 10 person-years), and external causes (such as injuries and poisonings; 12·8 days per 10 person-years). Being overweight (rate ratio [adjusted for age, sex, socioeconomic status, and chronic disease at baseline] 1·30, 95% CI 1·21–1·40; PAF_external_ 8·9%) and low physical activity (1·23, 1·14–1·34; 7·8%) were associated with absences due to musculoskeletal diseases; heavy episodic drinking (1·90, 1·41–2·56; 15·2%), smoking (1·70, 1·42–2·03; 11·8%), low physical activity (1·67, 1·42–1·96; 19·8%), and obesity (1·38, 1·11–1·71; 5·6%) were associated with absences due to depressive disorders; heavy episodic drinking (1·64, 1·33–2·03; 11·3%), obesity (1·48, 1·27–1·72; 6·6%), smoking (1·35, 1·20–1·53; 6·3%), and being overweight (1·20, 1·08–1·33; 6·2%) were associated with absences due to external causes; obesity (1·82, 1·40–2·36; 11·0%) and smoking (1·60, 1·30–1·98; 10·3%) were associated with absences due to circulatory diseases; low physical activity (1·37, 1·25–1·49; 12·0%) and smoking (1·27, 1·16–1·40; 4·9%) were associated with absences due to respiratory diseases; and obesity (1·67, 1·34–2·07; 9·7%) was associated with absences due to digestive diseases.

**Interpretation:**

Lifestyle factors are associated with sickness absence due to several diseases, but observational data cannot determine the nature of these associations. Future studies should investigate the cost-effectiveness of lifestyle interventions aimed at reducing sickness absence and the use of information on lifestyle for identifying groups at risk.

**Funding:**

NordForsk, British Medical Research Council, Academy of Finland, Helsinki Institute of Life Sciences, and Economic and Social Research Council.

## Introduction

The absence from work due to illness (ie, sickness absence) is a great concern in many countries, emphasising the importance of knowing the modifiable risk factor targets for disability prevention, maintaining an active work force, and extending working lives.^1^ Risk factors related to lifestyle, such as smoking, risky alcohol use, high body-mass index (BMI), and low physical activity, account for a substantial proportion of years of life lost due to disability and premature mortality.^2^ However, the importance of lifestyle factors in terms of sickness absence in working populations has rarely been in focus.^3^, ^4^, ^5^, ^6^

Available evidence on lifestyle and diagnosis-specific sickness absence is limited to investigations that have assessed one lifestyle factor or one diagnosis outcome at a time, thus preventing comparisons of the relative importance of different lifestyle factors for different diagnostic groups.^7^, ^8^, ^9^, ^10^, ^11^, ^12^, ^13^, ^14^, ^15^, ^16^, ^17^, ^18^, ^19^ Some of these studies have been small in scale (sample size between 200 and 4000 participants) and have provided imprecise estimates.^11^, ^12^, ^13^, ^14^, ^17^ The overall evidence is characterised by mixed findings.^7^, ^8^, ^9^, ^10^, ^11^, ^12^, ^13^, ^14^, ^15^, ^16^, ^17^, ^18^, ^19^

In this study, we aim to quantify the associations between lifestyle factors (smoking, alcohol consumption, BMI, and physical activity) and sickness absence due to six specific diagnoses: musculoskeletal diseases, depressive disorders, external causes (eg, injuries and poisonings), circulatory diseases, respiratory diseases, and digestive diseases.

**Research in context**

**Evidence before this study**

In addition to morbidity and mortality, lifestyle factors such as smoking, risky alcohol use, high body-mass index (BMI), and low physical activity might increase the risk of absence from work due to illness (ie, sickness absence). We searched PubMed for studies published before June 19, 2018, on lifestyle factors and sickness absence without language restrictions, by using the following search terms for titles and abstracts: (“physical” AND “activity” AND “sickness” AND “absence”) OR (“overweight” AND “sickness” AND “absence”) OR (“obesity” AND “sickness” AND “absence”) OR (“smoking” AND “sickness” AND “absence”) OR (“alcohol” AND “sickness” AND “absence”) OR (“health” AND “behaviours” AND “sickness” AND “absence”) OR (“lifestyle” AND “sickness” AND “absence”). We found evidence for an association between lifestyle factors and all-cause sickness absence, but findings from the 13 studies on diagnosis-specific sickness absence were mixed. Previous studies typically focused on a single lifestyle factor and outcome, applied heterogeneous methodologies, and included small sample sizes.

**Added value of this study**

In addition to quantifying the magnitude of the association between lifestyle factors and risk of diagnosis-specific sickness absence in a large multicohort study, we assessed the quality of the evidence against the following criteria: strength of association, consistency across cohorts, robustness to adjustments and multiple testing, and public health impact of the observed associations, assessed by population attributable fractions. We determined a high or moderate rating for the association of high BMI and low physical activity with musculoskeletal diseases; for heavy episodic drinking, smoking, high BMI, and low physical activity in relation to depressive disorders; and for heavy episodic drinking, smoking, and high BMI in relation to external causes. We also determined a high or moderate rating for the association of smoking and obesity with sickness absence due to circulatory diseases and of smoking and low physical activity with sickness absence due to respiratory diseases.

**Implications of all the available evidence**

This multicohort study shows that lifestyle-related factors are likely to be important for work capacity among the working-age population. If the associations were causal, removal of the lifestyle risk factors would result in a substantial reduction in sickness absence. Future studies should investigate the cost-effectiveness of lifestyle interventions aimed at reducing sickness absence and the use of information on lifestyle factors for identifying groups at risk.

## Methods

### Study population

We used individual-level data from four cohort studies: the Finnish Public Sector (FPS) study (Finland),^20^ which is an occupational cohort of public sector employees; the Health and Social Support (HeSSup) study (Finland),^21^ which is a population-based cohort of working-age adults; the Whitehall II study (UK), ^22^ a cohort of London-based civil servants; and the GAZEL study (France),^23^ a cohort of employees from the national gas and electricity company. All cohort studies were approved by local ethics committees: the Ethics Committee of the Hospital District of Helsinki and Uusimaa (Finland) approved FPS, the Turku University Central Hospital Ethics committee (Finland) approved HeSSup, the University College London Medical School committee on the ethics of human research (UK) approved Whitehall II, and the Inserm Ethics committee (France) approved GAZEL. All participants gave their informed consent.

We included respondents who were alive, responded to the baseline surveys, and were not retired from work before the start of follow-up of the respective studies. The FPS survey was done in 2004, HeSSup in 2003, GAZEL in 1997, and Whitehall II in 1991–94. Follow-up (time at risk) for all cohorts was until old-age pension, death, or end of follow-up period, whichever came first. Details of register follow-up are provided in the appendix (p 3). The mean follow-up was 6·5 (SD 1·4) years in FPS, 6·7 (1·1) in HeSSup, 3·8 (2·3) in GAZEL, and 4·4 (2·1) in Whitehall II.

### Procedures

Lifestyle factors were based on self-reported information, except for height and weight in one of the studies (Whitehall II). Smoking status included categories of current smoker and non-smoker. We defined high alcohol consumption as a weekly consumption exceeding 112 g of absolute alcohol for both men and women, according to 2016 guidelines in the UK.^24^ Moderate alcohol consumption referred to a weekly consumption of more than 0 g and 112 g or fewer. Heavy episodic drinking was defined as a participant reporting having passed out at least once because of heavy drinking during the past 12 months (data available in FPS and HeSSup). BMI was categorised as lower than 18·5 kg/m^2^ (underweight), 18·5–24·9 kg/m^2^ (normal weight), 25·0–29·9 kg/m^2^ (overweight), and 30·0 kg/m^2^ or higher (obesity). Leisure-time physical activity was a dichotomous variable, including low physical activity versus intermediate and high physical activity. Details of lifestyle factors are provided in the appendix (p 2).

Sickness absence was measured as the number of diagnosis-specific sickness absence days per year derived from health registers in all cohorts and summed up for the follow-up period, which was either until death, old-age pension, or the end of follow-up of the respective studies. Details of sickness absences and covariates in each cohort are provided in the appendix (pp 3–5).

### Statistical analysis

We examined the rate of diagnosis-specific sickness absence—the number of sickness absence days per year over the follow-up period—separately for six diagnoses: musculoskeletal diseases, depressive disorders, external causes (eg, injuries and poisonings), circulatory diseases, respiratory diseases, and digestive diseases. In the subgroup analyses of those who had at least 1 day of sickness absence, we examined the number of absence days per year recorded for these six conditions (indicating longer duration or repeated absences due to a diagnosis, eg, musculoskeletal disorder), and the number of absence days per year due to other causes (eg, absence days due to any of the other five conditions in addition to musculoskeletal disease).

We used a two-stage analysis. At the first stage, we analysed each cohort separately and used negative binomial regression analysis, which provides rate ratios and their 95% CIs for the number of sickness absence days and takes into account the length of follow-up (the offset variable in statistical models). Smokers were compared with non-smokers; participants with high alcohol consumption were compared with those with moderate alcohol consumption; participants in the overweight and obesity categories were each compared with those in the normal weight category; and participants with low physical activity were compared with those with high or moderate physical activity. The minimally adjusted models included age and sex as covariates. The multivariable models were also adjusted for socioeconomic status, chronic disease, and other lifestyle factors. In subgroup analyses, we stratified the analyses by prevalent chronic disease at baseline to assess whether the associations varied by chronic disease status. Study-specific results were analysed with SAS 9.4 software.

At the second stage, we pooled study-specific rate ratio (RR) estimates in a fixed effects meta-analysis, which is suitable for studies with few cohorts,^25^ with Stata 15 software. We examined heterogeneity in study-specific estimates with use of the *I*^2^ test. We used Bonferroni correction to compensate for multiple testing (a total of 36 tests from six lifestyle factor components and six sickness absence outcomes). We assessed the public health impact using population attributable fractions (PAF)—that is—the proportional reduction in diagnosis-specific sickness absence that would occur if exposure to a risk factor was reduced to an alternative ideal exposure scenario (eg, from obesity to normal weight).

To form a comprehensive summary of the findings, we assessed the evidence from the main analysis by rating the 36 observed estimates as high, moderate, or low or poor according to the following four criteria—strength of association: RR lower than 1·1 (low), 1·1–1·49 and significant (moderate), and 1·5 or higher (high); consistency: *I*^2^ values greater than 50% and significant (low), 25–50% (moderate), and lower than 25% (high); robustness to serial adjustments (plus exclusion of participants with a chronic disease) and multiple testing: RR not robust to adjustments (low), robust to adjustments, but not to multiple testing (moderate), and robust to adjustments and multiple testing (high); PAF: greater than 10% (high), 5–10% and significant (moderate), and lower than 5% (low). We considered strength of association by following Bradford Hill's criteria.^26^

### Role of the funding source

The funders of the study had no role in study design, data collection, data analysis, data interpretation, or writing of the report. MV, JP, and MKi had full access to all data. MV and MKi had final responsibility for the decision to submit for publication.

## Results

The baseline characteristics of the 74 296 participants (24 825 men and 49 471 women) are shown in table 1. About half of participants were overweight or obese, one in three to one in five were physically inactive, consumed high levels of alcohol, or both, one in five or fewer were smokers, and one in ten reported heavy episodic drinking. During 446 478 person-years at risk (mean follow-up of 6·0 years), the most common causes of sickness absence were musculoskeletal diseases, depressive disorders, external causes, and circulatory diseases.

**Table 1:** Characteristics of participants in four cohort studies

		**Finnish Public Sector study**^20^**(n=46 974)**	**Health and Social Support study**^21^**(n=12 056)**	**GAZEL study**^23^**(n=10 686)**	**Whitehall II study**^22^**(n=4580)**
Age, mean (SD)		45·5 (9·5)	41·8 (10·7)	51·0 (2·9)	48·7 (5·6)
Sex					
	Men	9231 (19·7)	4655 (38·6)	7645 (71·5)	3294 (71·9)
	Women	37 743 (80·4)	7401 (61·4)	3041 (28·5)	1286 (28·1)
Socioeconomic status					
	Low	7098 (15·1)	4483 (37·2)	1147 (10·7)	729 (15·9)
	Intermediate	12 990 (27·7)	3877 (32·2)	5559 (52·0)	2098 (45·8)
	High	26 886 (57·2)	3696 (30·7)	3980 (37·2)	1753 (38·3)
Chronic disease					
	No	27 554 (58·7)	7720 (64·0)	8747 (81·9)	3120 (68·1)
	Yes	19 420 (41·3)	4336 (36·0)	1939 (18·2)	1460 (31·9)
Smoking					
	No	37 870 (82·9)	9525 (79·8)	8622 (81·9)	3831 (85·4)
	Yes	7834 (17·1)	2415 (20·2)	1900 (18·1)	654 (14·6)
Alcohol consumption					
	No	6545 (14·0)	1565 (13·0)	1241 (11·9)	857 (18·7)
	Within recommended limits	32 278 (69·1)	7724 (64·2)	5619 (54·0)	2556 (55·8)
	High	7886 (16·9)	2740 (22·8)	3543 (34·1)	1165 (25·5)
Heavy episodic drinking*					
	No	43 283 (93·0)	10 598 (88·2)	..	..
	Yes	3234 (7·0)	1422 (11·8)	..	..
Body-mass index					
	Underweight	480 (1·1)	143 (1·2)	99 (0·9)	43 (1·0)
	Normal	24 196 (52·9)	6347 (53·0)	4997 (47·1)	2343 (52·4)
	Overweight	15 182 (33·2)	4068 (34·0)	4554 (42·9)	1664 (37·2)
	Obese	5911 (12·9)	1412 (11·8)	955 (9·0)	419 (9·4)
Low physical activity					
	No	37 565 (80·7)	9464 (78·9)	6266 (62·7)	3653 (79·8)
	Yes	8984 (19·3)	2538 (21·2)	3727 (37·3)	926 (20·2)
Participants with ≥1 day of sickness absence at follow-up					
	Musculoskeletal diseases	12 911 (27·5)	2165 (18·0)	1850 (17·3)	926 (20·2)
	Depressive disorders	3700 (7·9)	656 (5·4)	587 (5·5)	746 (16·3)
	External causes	5649 (12·0)	1139 (9·4)	1548 (14·5)	829 (18·1)
	Circulatory diseases	2490 (5·3)	440 (3·6)	628 (5·9)	113 (2·5)
	Respiratory diseases	3198 (6·8)	525 (4·4)	2127 (19·9)	2838 (62·0)
	Digestive diseases	2046 (4·4)	436 (3·6)	1135 (10·6)	617 (13·5)
Sickness absence days at follow-up, per 10 person-years					
	Musculoskeletal diseases	85·6	53·2	25·3	8·7
	Depressive disorders	30·2	18·1	21·3	13·3
	External causes	13·3	10·2	17·7	6·2
	Circulatory diseases	10·4	8·1	13·2	3·1
	Respiratory diseases	4·9	3·5	6·7	17·3
	Digestive diseases	3·0	2·0	6·1	2·7

Data are n (%), unless specified otherwise.

* Data available from Finnish Public Sector study^20^ and Health and Social Support study.^21^

**Figure 1:**
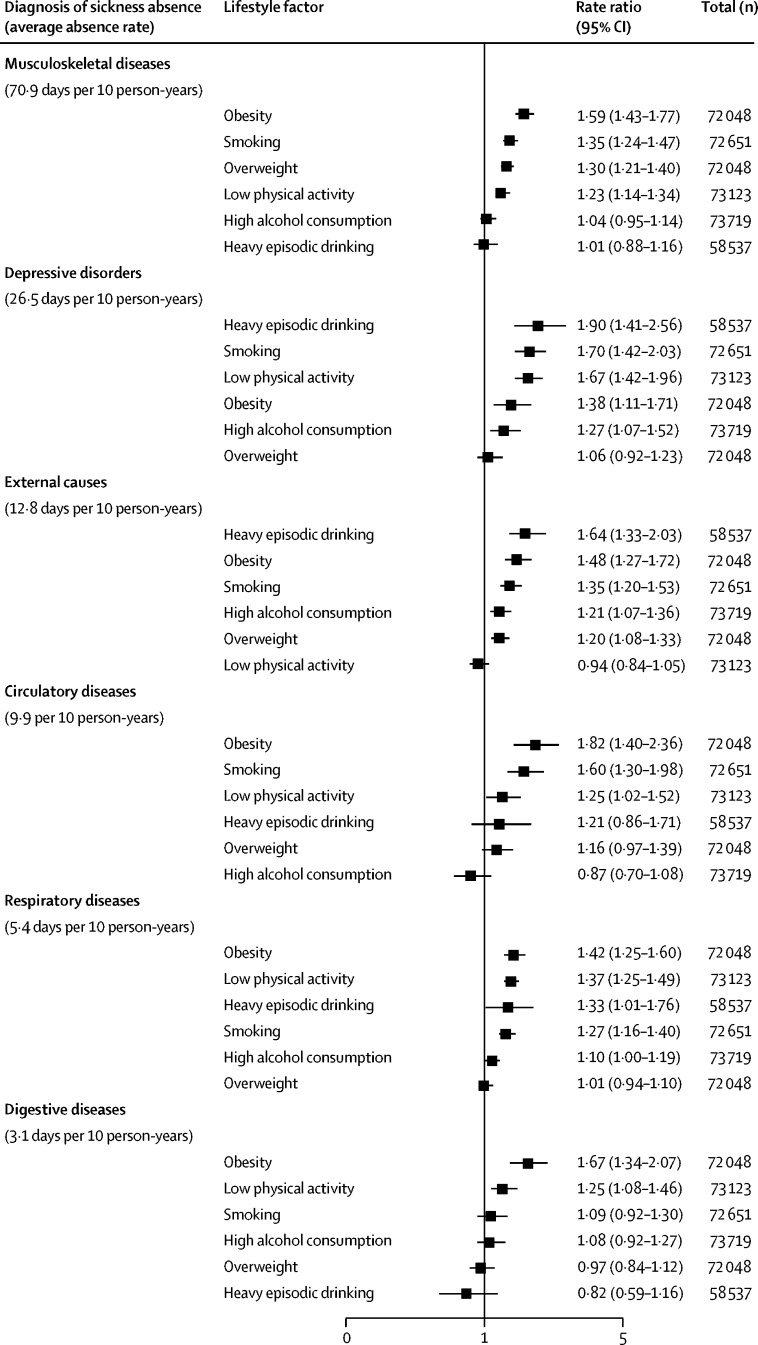
Rate ratio from meta-analyses for association between lifestyle factors and diagnosis-specific sickness absence, adjusted for age, sex, socioeconomic status and chronic disease Error bars denote 95% CI.

Figure 1 shows summary estimates for the association between lifestyle factors and diagnosis-specific sickness absence (study-specific results are available in the appendix, pp 7–12). All lifestyle factors were associated with musculoskeletal diseases, with the exception of high alcohol consumption and heavy episodic drinking. Likewise, all lifestyle factors were associated with sickness absence due to depressive disorders (with the exception of being overweight), external causes (with the exception of low physical activity), and respiratory diseases (with the exception of being overweight). Obesity, smoking, and low physical activity were associated with sickness absence due to circulatory diseases. Obesity and low physical activity alone were significantly associated with sickness absence due to digestive diseases. Most of these associations were robust to multivariable adjustments, and there were few differences in the results of subgroup analyses among participants with and without chronic disease at baseline (table 2). The PAF for each lifestyle factor and the diagnosis-specific sickness absence outcomes are shown in table 3.

**Table 2:** Multivariable adjusted summary estimates from meta-analyses of association between lifestyle factors and diagnosis-specific sickness absence

	**Age and sex adjusted (model 1)**	**Model 1 plus SES (model 2)**	**Model 2 plus adjustment for chronic disease and other lifestyle factors (model 3)**	**Model 2 excluding participants with a chronic disease (model 4)**	**Model 2 including only participants with a chronic disease (model 5)**
**Sickness absence due to musculoskeletal diseases**					
Smoking *vs* non-smoking	1·69 (1·54–1·85)	1·33 (1·21–1·45)	1·35 (1·23–1·48)	1·43 (1·26–1·63)	1·18 (1·04–1·34)
High *vs* moderate alcohol consumption	1·11 (1·02–1·22)	1·10 (1·01–1·21)	1·04 (0·95–1·14)	1·03 (0·91–1·17)	1·03 (0·90–1·18)
Heavy episodic drinking *vs* no episodic drinking	1·15 (1·00–1·33)	1·05 (0·91–1·21)	0·95 (0·82–1·09)	1·06 (0·86–1·31)	0·94 (0·78–1·13)
Overweight *vs* normal weight	1·46 (1·36–1·58)	1·37 (1·28–1·48)	1·37 (1·26–1·48)	1·29 (1·17–1·43)	1·31 (1·17–1·46)
Obesity *vs* normal weight	2·12 (1·90–2·37)	1·86 (1·67–2·07)	1·82 (1·62–2·03)	1·50 (1·27–1·77)	1·73 (1·50–1·98)
Low physical activity *vs* moderate and high activity	1·46 (1·34–1·59)	1·34 (1·23–1·45)	1·22 (1·12–1·34)	1·21 (1·07–1·36)	1·29 (1·16–1·44)
**Sickness absence due to depressive disorders**					
Smoking *vs* non-smoking	1·93 (1·61–2·31)	1·95 (1·63–2·33)	1·93 (1·59–2·33)	1·76 (1·37–2·27)	1·63 (1·27–2·09)
High *vs* moderate alcohol consumption	1·27 (1·07–1·51)	1·30 (1·09–1·55)	1·06 (0·88–1·28)	1·37 (1·07–1·74)	1·09 (0·84–1·40)
Heavy episodic drinking *vs* no episodic drinking	1·85 (1·37–2·50)	1·77 (1·30–2·39)	1·79 (1·31–2·44)	2·28 (1·38–3·76)	1·38 (0·95–2·00)
Overweight *vs* normal weight	1·22 (1·05–1·41)	1·19 (1·03–1·38)	1·18 (1·01–1·37)	1·08 (0·87–1·33)	1·13 (0·91–1·40)
Obesity *vs* normal weight	1·61 (1·30–2·00)	1·63 (1·32–2·03)	1·62 (1·29–2·03)	1·51 (1·07–2·12)	1·26 (0·96–1·65)
Low physical activity *vs* moderate and high activity	1·78 (1·51–2·10)	1·73 (1·47–2·04)	1·52 (1·27–1·81)	1·63 (1·29–2·07)	1·57 (1·26–1·96)
**Sickness absence due to external causes**					
Smoking *vs* non-smoking	1·53 (1·35–1·73)	1·35 (1·19–1·53)	1·34 (1·17–1·52)	1·40 (1·19–1·64)	1·26 (1·03–1·54)
High *vs* moderate alcohol consumption	1·18 (1·04–1·33)	1·22 (1·08–1·37)	1·19 (1·05–1·36)	1·19 (1·02–1·38)	1·20 (0·98–1·47)
Heavy episodic drinking *vs* no episodic drinking	1·75 (1·42–2·17)	1·68 (1·36–2·07)	1·60 (1·28–1·98)	1·61 (1·20–2·15)	1·65 (1·21–2·24)
Overweight *vs* normal weight	1·28 (1·15–1·42)	1·21 (1·09–1·34)	1·24 (1·11–1·38)	1·22 (1·07–1·39)	1·12 (0·94–1·33)
Obesity *vs* normal weight	1·70 (1·46–1·98)	1·57 (1·35–1·83)	1·57 (1·34–1·83)	1·54 (1·25–1·91)	1·34 (1·07–1·66)
Low physical activity *vs* moderate and high activity	1·04 (0·93–1·16)	0·98 (0·88–1·10)	0·86 (0·76–0·97)	0·91 (0·79–1·06)	0·93 (0·78–1·11)
**Sickness absence due to circulatory diseases**					
Smoking *vs* non-smoking	1·70 (1·38–2·10)	1·59 (1·28–1·97)	1·64 (1·32–2·05)	1·71 (1·28–2·30)	1·55 (1·13–2·13)
High *vs* moderate alcohol consumption	0·94 (0·76–1·16)	0·91 (0·74–1·13)	0·87 (0·69–1·09)	0·82 (0·61–1·10)	1·00 (0·72–1·37)
Heavy episodic drinking *vs* no episodic drinking	1·27 (0·90–1·78)	1·27 (0·90–1·79)	1·18 (0·83–1·69)	1·13 (0·70–1·84)	1·23 (0·76–1·99)
Overweight *vs* normal weight	1·38 (1·15–1·64)	1·29 (1·08–1·55)	1·32 (1·10–1·59)	1·07 (0·84–1·37)	1·51 (1·14–1·99)
Obesity *vs* normal weight	2·24 (1·73–2·89)	2·11 (1·63–2·73)	2·05 (1·57–2·68)	1·94 (1·31–2·87)	2·06 (1·46–2·90)
Low physical activity *vs* moderate and high activity	1·46 (1·20–1·78)	1·37 (1·13–1·67)	1·22 (0·99–1·50)	1·16 (0·89–1·53)	1·33 (1·00–1·77)
**Sickness absence due to respiratory diseases**					
Smoking *vs* non-smoking	1·41 (1·28–1·56)	1·28 (1·16–1·42)	1·29 (1·17–1·43)	1·16 (1·03–1·29)	1·52 (1·23–1·86)
High *vs* moderate alcohol consumption	1·08 (0·99–1·18)	1·14 (1·04–1·25)	1·15 (1·05–1·27)	0·97 (0·88–1·07)	1·53 (1·26–1·86)
Heavy episodic drinking *vs* no episodic drinking	1·45 (1·10–1·93)	1·45 (1·09–1·92)	1·27 (0·96–1·69)	1·23 (0·81–1·86)	1·48 (1·00–2·19)
Overweight *vs* normal weight	1·07 (0·99–1·16)	1·02 (0·95–1·11)	1·04 (0·96–1·13)	1·06 (0·97–1·16)	0·92 (0·78–1·10)
Obesity *vs* normal weight	1·56 (1·38–1·77)	1·53 (1·35–1·73)	1·45 (1·28–1·65)	1·39 (1·20–1·61)	1·47 (1·17–1·84)
Low physical activity *vs* moderate and high activity	1·68 (1·54–1·84)	1·42 (1·30–1·55)	1·33 (1·21–1·46)	1·38 (1·25–1·52)	1·38 (1·15–1·65)
**Sickness absence due to digestive diseases**					
Smoking *vs* non-smoking	1·30 (1·09–1·54)	1·13 (0·96–1·35)	1·18 (0·99–1·41)	0·99 (0·80–1·22)	1·35 (1·01–1·82)
High *vs* moderate alcohol consumption	1·10 (0·94–1·30)	1·09 (0·93–1·28)	1·05 (0·89–1·24)	1·09 (0·89–1·32)	0·99 (0·74–1·32)
Heavy episodic drinking *vs* no episodic drinking	0·89 (0·63–1·25)	0·85 (0·60–1·19)	0·80 (0·56–1·14)	0·79 (0·49–1·28)	0·84 (0·51–1·37)
Overweight *vs* normal weight	1·08 (0·93–1·24)	0·99 (0·86–1·15)	1·01 (0·87–1·17)	0·99 (0·83–1·18)	0·94 (0·73–1·22)
Obesity *vs* normal weight	1·94 (1·56–2·42)	1·75 (1·41–2·17)	1·73 (1·38–2·17)	1·77 (1·32–2·37)	1·37 (0·98–1·92)
Low physical activity *vs* moderate and high activity	1·30 (1·11–1·51)	1·29 (1·11–1·50)	1·15 (0·98–1·35)	1·28 (1·06–1·54)	1·12 (0·87–1·45)

Data are rate ratio (95% CI) adjusted for each model. Sensitivity analyses (models 4 and 5) were stratified by chronic disease at baseline. Results for models adjusted for age, sex, socioeconomic status (SES), and chronic disease are provided in figure 1.

**Table 3:** Population attributable fraction (PAF) for diagnosis-specific sickness absence adjusted for age, sex, socioeconomic status, and chronic disease

	**Prevalence**	**Musculoskeletal diseases**	**Depressive disorders**	**External causes**	**Circulatory diseases**	**Respiratory diseases**	**Digestive diseases**
**Smoking**							
Current data	17·6%	5·8 (4·1 to 7·6)	11·0 (6·9 to 15·4)	5·8 (3·4 to 8·5)	9·6 (5·0 to 14·7)	4·5 (2·7 to 6·6)	1·6 (−1·4 to 5·0)
Prevalence from external source	19·2%	6·3 (4·4 to 8·3)	11·8 (7·5 to 16·5)	6·3 (3·7 to 9·2)	10·3 (5·4 to 15·8)	4·9 (3·0 to 7·1)	1·7 (−1·6 to 5·4)
High alcohol consumption*							
Current data	20·8%	0·8 (−1·0 to 2·7)	5·1 (1·4 to 9·0)	4·2 (1·5 to 6·8)	−2·7 (−6·8 to 1·5)	1·9 (0·0 to 3·5)	1·6 (−1·7 to 4·9)
Prevalence from external source	30·5%	1·2 (−1·5 to 3·9)	7·2 (2·0 to 12·5)	6·0 (2·1 to 9·6)	−4·1 (−10·3 to 2·2)	2·8 (0·0 to 5·0)	2·3 (−2·5 to 7·0)
Heavy episodic drinking*							
Current data	8·0%	0·1 (−1·0 to 1·3)	6·7 (3·2 to 11·1)	4·9 (2·6 to 7·6)	1·7 (−1·1 to 5·4)	2·6 (0·1 to 5·7)	−1·5 (−3·4 to 1·3)
Prevalence from external source	19·9%	0·2 (−2·4 to 3·1)	15·2 (7·5 to 23·7)	11·3 (6·2 to 17·0)	4·0 (−2·9 to 12·4)	6·2 (0·2 to 13·1)	−3·7 (−8·9 to 3·1)
**Overweight**†							
Current data	35·0%	8·9 (6·5 to 11·4)	2·0 (−2·8 to 6·9)	6·2 (2·6 to 9·6)	4·8 (−1·0 to 10·5)	0·3 (−2·1 to 3·2)	−1·0 (−5·7 to 3·6)
Prevalence from external source	35·7%	8·9 (6·6 to 11·3)	2·0 (−2·9 to 6·9)	6·2 (2·7 to 9·6)	4·8 (−1·0 to 10·3)	0·3 (−2·1 to 3·2)	−1·0 (−5·7 to 3·5)
**Obesity**†							
Current data	11·9%	6·0 (4·6 to 7·5)	4·3 (1·3 to 7·3)	5·1 (3·0 to 7·2)	8·5 (4·6 to 12·5)	4·8 (3·0 to 6·5)	7·5 (4·1 to 10·9)
Prevalence from external source	15·9%	7·8 (6·0 to 9·7)	5·6 (1·8 to 9·4)	6·6 (4·0 to 9·3)	11·0 (6·0 to 16·0)	6·2 (3·9 to 8·4)	9·7 (5·4 to 14·0)
**Low physical activity**							
Current data	22·4%	4·9 (3·0 to 7·1)	13·1 (8·6 to 17·7)	−1·4 (−3·7 to 1·1)	5·3 (0·4 to 10·4)	7·7 (5·3 to 9·9)	5·3 (1·8 to 9·3)
Prevalence from external source	36·8%	7·8 (4·9 to 11·1)	19·8 (13·4 to 26·1)	−2·3 (−6·3 to 1·8)	8·4 (0·7 to 16·1)	12·0 (8·4 to 15·3)	8·4 (2·9 to 14·5)

Data are PAF (95% CI) for sickness absence by diagnosis. Prevalence of risk factors from an external source was obtained for the general population from EUROSTAT 2014 (28 EU countries) for overweight (body-mass index 25–29·9 kg/m^2^), obesity (≥30 kg/m^2^), smoking (daily), and heavy episodic drinking (at least once every month); *Lancet Global Health* 2018 study of high-income countries for physical inactivity (not doing at least 150 min of moderate intensity or 75 min of vigorous intensity physical activity per week, or any equivalent combination of the two); *Lancet* 2018 study of 22 centres in ten European countries for high alcohol consumption (>100 g absolute alcohol per week; references in the appendix).

* PAF for the reduction in sickness absence if people who reduced their high alcohol consumption consumed moderate levels of alcohol (rather than becoming abstainers); abstainers (prevalence 13·8% in current data and 15·2% in external sources) were taken into account in PAF analyses, but were not included in the reference group consisting of participants with moderate alcohol consumption.

† Participants who were underweight (<18·5 kg/m^2^) were excluded from this analysis.

**Figure 2:**
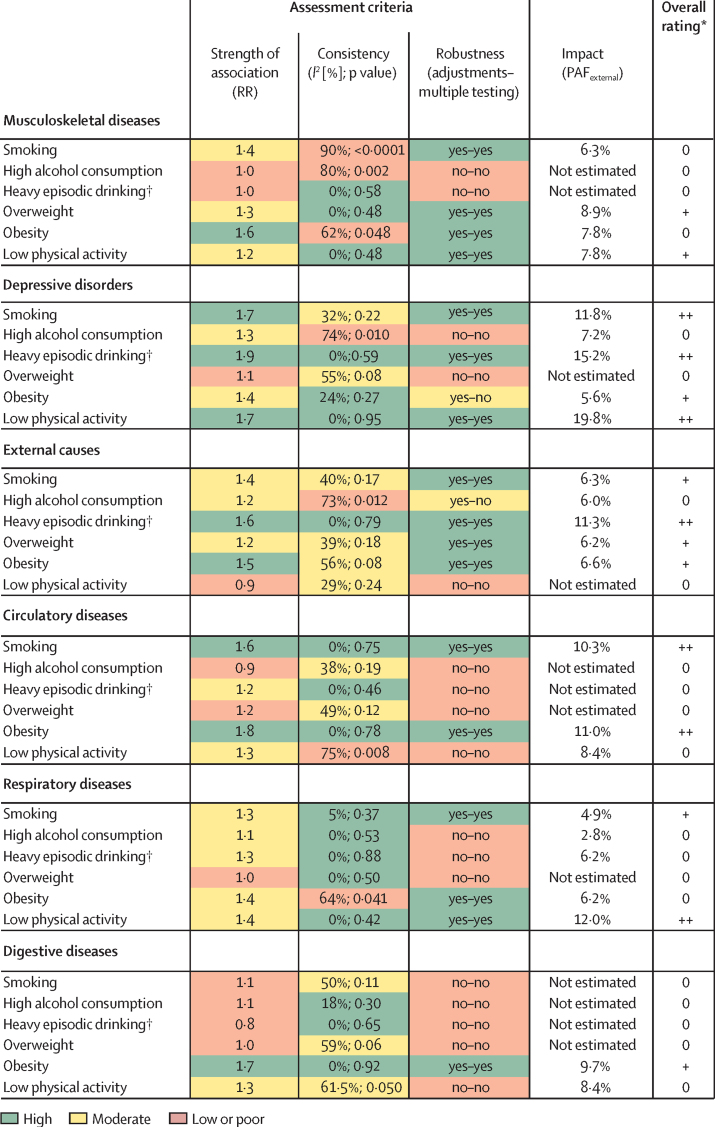
Heat map of evidence of association between lifestyle factors and diagnosis-specific sickness absence Strength of association: rate ration (RR) lower than 1·1 (low), 1·1–1·49 and significant (moderate), and 1·5 or higher and significant (high). Consistency: *I*^2^ values greater than 50% and significant (low), 25–50% (moderate), and lower than 25% (high). Robustness to serial adjustments and multiple testing: RR not robust to adjustments (low); robust to adjustments, but not to multiple testing (moderate); and robust to adjustments and multiple testing (high). Population attributable fractions (PAF) on the basis of exposure prevalence estimates obtained from European countries (PAF_external_): greater than 10% (high), 5–10% and significant (moderate), and lower than 5% (low). Although causal associations can be strong and weak, strong multivariable-adjusted associations are less likely to be confounded than weak associations. For example, an RR of 1·3 between a single confounder and sickness absence could explain a weak 1·05 times increase in risk of sickness absence associated with the lifestyle factor; the corresponding RR required to explain a strong 1·5 times increased association between the lifestyle factor and sickness absence would be as high as 2·4. Details of PAF calculations are provided in the appendix (p 6). *Overall rating is indicated as: 0 (at least one low rating in strength of association, consistency, or robustness), + (high or moderate rating for strength of association, consistency and robustness, and moderate PAF); or ++ (high or moderate rating for strength of association, consistency and robustness, and high PAF). †Data available from Finnish Public Sector study^20^ and Health and Social Support study.^21^ Not estimated=non-significant association or negative PAF.

By using the four criteria to assess and rate the overall evidence for each association between lifestyle factors and diagnosis-specific sickness absence (figure 2), we determined a high rating, with PAFs higher than 10%, for the associations of smoking, heavy episodic drinking, and low physical activity with absence due to depressive disorders; between heavy episodic drinking and absence due to external causes; between smoking and obesity, and absence due to circulatory diseases; and between low physical activity and absence due to respiratory diseases. We determined a moderate rating (PAFs ≤10%) for several other associations between lifestyle factors and diagnosis-specific sickness absences (figure 2).

Subgroup analyses included participants with 1 or more sickness absence days from each diagnostic group. The results are shown in the appendix (pp 13–14) and largely replicate those in the main analysis—in the assessment of both the number of sickness absence days within a single diagnosis and the number of sickness absence days due to other reasons. Sensitivity analyses, with the exclusion of the Whitehall and GAZEL studies, suggested no major differences in the results despite the variation in sickness absence measures (appendix, pp 15–16).

## Discussion

Our findings, from more than 74 000 employees from the UK, France, and Finland, showed that lifestyle factors can be associated with higher diagnosis-specific sickness absence rates. In terms of effect size, consistency, robustness, and PAF for sickness absence, we determined a high or moderate rating for the association of high BMI and low physical activity with musculoskeletal diseases; for heavy episodic drinking, smoking, high BMI, and low physical activity in relation to depressive disorders; and for heavy episodic drinking, smoking, and high BMI in relation to external causes. We also determined a high or moderate rating for the association of smoking and obesity with sickness absence due to circulatory diseases and of smoking and low physical activity with sickness absence due to respiratory diseases.

Previous findings regarding the association between high BMI and absence due to musculoskeletal diseases have been inconsistent,^13^, ^15^, ^17^ but there are plausible mechanisms for this association, because high BMI might contribute to pain and limitations in mobility, which restrict work.^27^ Also, physical activity might protect employees from absence due to musculoskeletal diseases.^10^ These findings are plausible because physical activity has many beneficial effects on muscle strength and mobility. Although smoking provided a high summary estimate for musculoskeletal-related absences, the findings suffered from heterogeneity between cohorts, which has also been the case in previous smaller studies.^13^, ^15^, ^17^

Low physical activity, smoking, heavy episodic drinking, and high BMI were associated with sickness absence due to depressive disorders. Our findings add evidence to previous research and treatment guidelines, which suggest that targeting physical activity might reduce the burden associated with depressive disorders.^28^ Smoking is associated with adverse changes in brain structure and neural circuity in the brain regions implicated in many mental disorders^29^ and with neuroadaptations in the nicotinic pathways in the brain, which manifest as depressed mood, agitation, and anxiety shortly after a cigarette is smoked.^30^ Despite these plausible mechanisms, a mendelian randomisation study^31^ suggested that the link between smoking and depression might not be causal. The observed association between heavy episodic drinking and sickness absence due to depressive disorders is in accordance with previous findings on high alcohol consumption and mental disorder-related sickness absence,^11^ although our study was probably the first to assess the association between heavy episodic drinking and depressive disorders. This finding might be explained by at least three possible pathways: a direct causal pathway; a so-called self-medication hypothesis (in which a pre-existing depressive disorder increases alcohol consumption); or a bidirectional or reciprocal causal relationship or causation by another mechanism (such as genetic vulnerability).^32^ For high BMI and depressive disorders, the association might also be bi-directional.^33^ However, the pathways linking obesity to depressive disorders have been suggested to be related to inflammatory factors, dysregulation of the hypothalamic-pituitary-adrenal axis, and metabolic and psychosocial factors.^33^

Regarding sickness absence due to external causes, such as injuries and poisonings, our evidence emphasised associations with smoking, heavy episodic drinking, and high BMI. Smoking and heavy episodic drinking might reflect a risk-taking lifestyle, which increases the risk of accidents and injuries. Previous research has shown an association between smoking and occupational injuries.^34^ This link might be explained by, for example, smoking-induced disturbed sleep and other conditions that affect physical functions, which can increase the risk of injury. Our results are also in accordance with a 2017 review^32^ that suggested a causal association between high alcohol consumption and injuries. The risk has been shown to be directly related to blood alcohol concentration: the higher the concentration, the higher the risk.^32^ Other evidence suggests that interventions targeted at people with a drinking problem can reduce the risk of injuries.^35^ Our findings on the association between high BMI and sickness absence due to external causes add to a previous study using FPS data, in which the association between BMI and recorded occupational injuries was used as an outcome, instead of sickness absence.^36^ The underlying mechanisms might relate to overall fatigue, disturbed sleep, limited mobility, and medications for chronic diseases, which are all more common among people with high BMI.

We rated the evidence of smoking and high BMI in relation to the risk of sickness absence due to circulatory diseases as strong, in line with the established evidence on morbidity, mortality, and disability-adjusted life-years.^2^, ^37^ Our study adds to previous research by showing the associations with incapacity to work among working populations. Smoking adversely influences the circulatory system at all major stages of atherosclerosis, increases the likelihood of pathological atherothrombosis formation, and enhances inflammation and blood clotting.^38^ The mechanisms linking high BMI to circulatory and several other diseases include health behaviours, adverse physiological and anatomical changes, inflammation, and cardiovascular and metabolic dysregulation.^27^

We are not aware of previous studies on the association between low physical activity and sickness absence due to respiratory disease, but our findings regarding smoking correspond to the results from a study^16^ of more than 5000 assistant nurses, whereas two other smaller-scale studies on the same subject^12^, ^14^ reported no association. This positive association is as expected, given the hazardous load of smoking on the respiratory system. Physical activity, in turn, increases cardiorespiratory fitness,^28^ which can protect from respiratory diseases.

To our knowledge, there are no previous studies on the association between obesity and sickness absence due to digestive diseases, but our findings are in line with previous evidence on obesity being a risk factor for digestive morbidity.^39^ Specific diseases that are related to obesity include oesophageal diseases, gastritis, diarrhoea, diverticular diseases, and gallbladder problems. The mechanisms might relate to low-grade chronic systemic inflammation, changes in metabolism, and the distribution of adipose tissue in the abdomen.^39^ In our study, neither high-volume alcohol consumption nor heavy episodic drinking were associated with sickness absences due to digestive diseases, although there is evidence that alcohol consumption is a risk factor for digestive diseases.^32^ This discrepancy requires future studies to be done with a more detailed analysis of alcohol consumption.

According to our PAF results, the greatest public health benefits in terms of reduced sickness absence due to depressive disorders were related to the elimination of low physical activity, heavy episodic drinking, and smoking. Additionally, increasing physical activity would reduce sickness absence due to respiratory diseases, quitting smoking and reducing obesity would reduce sickness absence due to circulatory diseases, and quitting heavy episodic drinking would reduce sickness absence due to external causes. However, these findings should be interpreted cautiously, because the underlying assumption for PAF is that the observed association is causal and that a perfect intervention can eradicate the risk factor. Complete removal of lifestyle risk factors from the working population is unrealistic; thus, all PAF reported in this study are likely to be overoptimistic.

Our subgroup analyses examined lifestyle as a predictor of sickness absence among employees on sick leave. Lifestyle factors, particularly obesity, smoking, and low physical activity, were associated with longer or more frequent absences and multiple-cause absences at follow-up. We observed fewer sickness absence days among participants who were overweight and had respiratory or digestive diseases than among those whose BMI was within the normal range. This difference might reflect weight loss associated with more severe forms of these diseases. Our findings on sickness absence due to multiple diagnoses suggest that, when treating an individual with sickness absence due to a specific disease, attention should also be paid to lifestyle factors that seem to increase the risk of sickness absence due to multiple causes. To date, there are very few intervention studies in this field and little evidence on the effect of lifestyle interventions on absence days.^40^, ^41^ Another relevant topic for future studies involves the interactions between chronic diseases and lifestyle factors. Such research should include an analysis of disease trajectories and an examination of diseases that are likely to be linked to each other.

Common limitations in meta-analyses are heterogeneity between studies and small estimates that are not robust to adjustment for confounding. To take these limitations into account, we set a conservative criteria for what we defined as strong support: a strong association, consistency across studies, and robustness to adjustments and multiple testing. Consistency is important, because homogeneous study-specific estimates provide assurance that the association observed in a new study will be similar. By contrast, if the heterogeneity in study-specific estimates is high, unless the reasons for heterogeneity are well understood, the association in a new study will be hard to predict. For example, although the summary RRs for the associations between obesity and sickness absence due to musculoskeletal and respiratory diseases were high, they were not considered robust because of high heterogeneity in study-specific estimates. Regarding internal validity, bias and confounding are present to some degree in all observational studies. We did analyses with mutual adjustments for lifestyle factors to address the confounding that arises from the clustering of unhealthy lifestyles into the same individuals. We excluded participants with a chronic disease to reduce bias due to reverse causation (eg, ill health can limit physical activities) and examined the associations among those with chronic disease to assess whether the associations vary by chronic disease status. We used multiple-comparison corrections to determine statistical significance of the observed associations in a context of multiple testing. Our main analyses, adjusted for chronic diseases, represent a conservative approach. Chronic diseases are potential confounders because they affect lifestyle factors, but they are also on the pathway from lifestyle factors to increased sickness absence. We observed no major differences in the results regarding participants with and without chronic disease, which suggests that over-adjustment as a result of treating chronic disease as a covariate is an unlikely source of major bias in this study. Despite all these measures, our findings should be interpreted cautiously, because residual confounding due to unmeasured factors and bias due to reverse causality might have led to overestimation or underestimation of associations.

Further limitations to this study include self-reported measurements of lifestyle factors and some of the covariates, crude measurements of heavy episodic drinking and physical activity, and the fact that we compared current smokers with non-smokers, which was a heterogeneous group that included both ex-smokers and people who had never smoked. We did not assess smoking intensity, which might have yielded stronger associations for smokers who smoked more.^42^ Because people tend to under-report their alcohol use, some of the risky drinkers might have been wrongly assigned to the moderate drinkers group, which might have diluted the estimates. The study populations consisted of employed men and women, among whom the prevalence of severe alcohol use disorders is likely to be low, as was shown in a Finnish population-based study (5·2%).^11^

Moreover, the sickness absence data differed between countries. In Finland (FPS^20^ and HeSSup^21^ studies), diagnosis-specific sickness absence episodes are only registered for absences lasting longer than 9 days, including absences with very long duration (rehabilitation and disability allowances). In the GAZEL^23^ and Whitehall II^22^ cohorts, sickness absence registers also included short-term absences. However, in GAZEL, the coverage of diagnoses in absences shorter than 7 days was about 50%.^8^ Shorter absences are often caused by respiratory diseases (common cold and other acute upper respiratory infections), many of which were missed when monitoring longer absence episodes alone. In FPS, HeSSup, and GAZEL cohorts, sickness absence data included medically certified data, whereas in the Whitehall II cohort, it was a mixture of records from self-certified absences lasting 1 to 7 days and medically-certified absences exceeding 7 days. Further differences included those related to the diagnostic systems used (International Classification of Diseases [ICD] 10 in FPS, HeSSup, and GAZEL, but ICD-8 in Whitehall II). However, in a sensitivity analysis excluding the Whitehall II and GAZEL cohorts one at a time, the results remained largely unchanged, suggesting that variation in the data was not a major source of bias.

In conclusion, this multicohort study suggests that lifestyle-related factors are important for an individual's capacity to work during an age range when the onset of major non-communicable diseases, permanent disability, and death are still very rare.^2^ Future studies should investigate the cost-effectiveness of lifestyle interventions aimed at reducing sickness absence and the use of lifestyle information for identifying groups at risk.

For the **Whitehall II data sharing policy** see www.uc.ac.uk/whitehallII/data-sharing

For the **GAZEL data sharing policy** see https://openbioresources.metajnl.com/articles/10.5334/ojb.ac/

## Data sharing
