# Supplementary material for: Lifestyle factors and risk of sickness absence from work: a multicohort study
Source: Lancet Public Health. 2018 Nov 5;3(11):e545–54. doi: 10.1016/S2468-2667(18)30201-9 (PMC6220357; doi:10.1016/S2468-2667(18)30201-9)
Supplement: Supplementary appendix [file mmc1.pdf]

THE LANCET
Public Health

Supplementary appendix

This appendix formed part of the original submission and has been peer reviewed. We post it as supplied by the authors.

Supplement to: Virtanen M, Ervasti J, Head J, et al. Lifestyle factors and risk of sickness absence from work: a multicohort study. *Lancet Public Health* 2018; **3:** e545–54.

Supplementary appendix

Supplement to: Virtanen M, Ervasti J, Head J, et al. Lifestyle and risk of absence from work due to specific diagnoses: A multi-cohort study. *Lancet Public Health*

Contents **Page**

Description of lifestyle factors 2

Description of sickness absence 3

Description of covariates 5

Description of Population Attributable fraction (PAF) 6

Supplementary Table 1: Rate ratio (RR) and 95% confidence intervals (CI) for association between lifestyle factors and sickness absence due to musculoskeletal diseases in each study cohort 7

Supplementary Table 2: Rate ratio (RR) and 95% confidence intervals (CI) for association between lifestyle factors and sickness absence due to depressive disorders in each study cohort 8

Supplementary Table 3: Rate ratio (RR) and 95% confidence intervals (CI) for association between lifestyle factors and sickness absence due to external causes in each study cohort 9

Supplementary Table 4: Rate ratio (RR) and 95% confidence intervals (CI) for association between lifestyle factors and sickness absence due to circulatory diseases in each study cohort 10

Supplementary Table 5: Rate ratio (RR) and 95% confidence intervals (CI) for association between lifestyle factors and sickness absence due to respiratory diseases each study cohort 11

Supplementary Table 6: Rate ratio (RR) and 95% confidence intervals (CI) for association between lifestyle factors and sickness absence due to digestive diseases in each study cohort 12

Supplementary Figure 1: Rate ratio (RR) and 95% confidence intervals (CI) from meta-analyses for association between lifestyle factors and diagnosis-specific sickness absence in the subgroups of sick-listed participants 13

Supplementary Figure 2: Rate ratio (RR) and 95% confidence intervals (CI) from meta-analyses for association between lifestyle factors and diagnosis-specific sickness absence in the subgroups of sick-listed participants 14

Supplementary Table 7: Summary estimates from meta-analyses for association between lifestyle factors and sickness absence days due to specific diagnoses in the total population of three cohort studies (FPS, HeSSup, Gazel), excluding the Whitehall II cohort 15

Supplementary Table 8: Summary estimates from meta-analyses for association between lifestyle factors and sickness absence days due to specific diagnoses in the total population of three cohort studies (FPS, HeSSup, Whitehall), excluding the GAZEL cohort 16

Description of lifestyle factors

Smoking was based on self-reports in all cohorts, and was dichotomized into current smokers or non-smokers. Alcohol use was based on self-reported average weekly consumption. We defined high alcohol consumption as weekly consumption exceeding 112g of absolute alcohol for both men and women, according to the most recent guidelines in the United Kingdom.[1] Moderate alcohol consumption referred to >0g and ≤112g/week. Heavy episodic drinking was defined if a participant reported having passed out at least once due to heavy drinking during the past 12 months (available in FPS and HeSSup study cohorts). Body mass index (BMI) was based on self-reported height and weight in FPS, HeSSup, and GAZEL and measured at the study clinic in the Whitehall II study. Leisure-time physical activity was based on self-reports in all cohorts. In FPS and HeSSup, physical activity was based on questions about average hours per week spent briskly walking, jogging or running, and low physical activity was defined as less than 0.5 hour of each. In GAZEL, physical activity was based on a question about practising any sport, from which the responses 'yes, competitively', 'yes, regularly', and 'yes, occasionally' indicated intermediate/high activity and 'no' indicated low physical activity. In Whitehall II, participants were asked about the duration and frequency per week spent doing light, moderate and vigorous intensity physical activity. Physical activity was then categorised as high activity (≥2.5 hours/week of moderate or ≥1 hour/week of vigorous physical activity), low activity (<1 hour/week of moderate and <1 hour/week of vigorous physical activity) or intermediate activity (if not active or inactive).[2]

References

1. UK Chief Medical Officers. UK Chief Medical Officers' Low Risk Drinking Guidelines 20162016. https://www.gov.uk/government/publications/alcohol-consumption-advice-on-low-risk-drinking (accessed September 9, 2018).
2. Stringhini S, Sabia S, Shipley M, et al. Association of socioeconomic position with health behaviors and mortality. *JAMA* 2010; **303**(12): 1159-66.

Description of sickness absence

Sickness absence was measured as the number of diagnosis-specific sickness absence days during the follow-up period, derived from health registers in all cohorts. In FPS and HeSSup, register information on the dates of medically-certified sickness absence (sick leave, rehabilitation and disability allowance) exceeding nine days was available from the Social Insurance Institution of Finland and the Finnish Centre for Pensions and merged into single datasets. These institutions do not collect data on shorter absences. These were followed up from January 1, 2004 to December 31, 2010 in HeSSup and from January 1, 2005 to December 31, 2011 in FPS. In GAZEL, the information on annual days of medically certified sickness absence was obtained from the employer's records for a follow-up period from January 1, 1998 to December 31, 2004. In GAZEL, a medical certificate was required from day 1, and the data included all episodes of sickness absence irrespective of length. However, the cause of sickness absence was missing for 50% of absences of fewer than 7 days.[1] In Whitehall II, information on all sickness absence was obtained from the Civil Service (employer) records for those (93%) employees who gave consent to have their sickness absence monitored, for a follow-up period from 1991-1994 (survey wave) until the end of 1998. In Whitehall II, the sickness absence data included both short-term (1-7 days) and long-term (>7 days) absences. The cause of absence was self-reported in shorter absences whereas for absences longer than seven calendar days, a medical certificate was required. In all cohorts, the sickness absence data included start and end dates for each absence episode. From this information, we calculated the number of diagnosis-specific days for the whole follow-up, which was until either death, old-age pension, or the end of follow-up. In the Whitehall II and GAZEL cohort studies, which relied on the sickness absence records obtained from employer's registers, follow-up was terminated if the participant changed jobs.

Diagnoses of sickness absence were coded according to ICD-10[2] in HeSSup, GAZEL, and FPS. We used codes F00-F99 to define mental and behavioural disorder-related sickness absences; I00-I99 to define sickness absences due to diseases of the circulatory system; J00-J99 to define sickness

absences due to diseases of the respiratory system; K00-K93 to define sickness absences due to diseases of the digestive system; M00-M99 to define sickness absences due to diseases of the musculoskeletal system and connective tissue; and S00-T98 to define sickness absences due to injury, poisoning and certain other consequences of external causes. In Whitehall II, diagnoses were recorded by the civil service using a detailed coding system (586 possible codes) adapted from the ICD-8 classification. These codes were converted to a smaller number of disease categories using the morbidity coding system of the Royal College of General Practitioners (RCGP).[3] For this study, we used the following RCGP categories 'Diseases of the musculoskeletal system and connective tissue', 'Diseases of digestive system', 'Diseases of respiratory system', and 'Injury and Poisoning'. We combined the categories 'Cardiovascular', 'Cerebrovascular' and 'Peripheral vascular diseases' to define sickness absences due to diseases of the circulatory system. For mental health, we included the category 'Mental disorders'(except psychosis) plus some additional mental health related codes from the RCGP category 'Symptoms and Ill-defined conditions' such as 'stress'.[3]

References

1. Morois S, Airagnes G, Lemogne C, et al. Daily alcohol consumption and sickness absence in the GAZEL cohort. *Eur J Public Health* 2017; **27**: 482-8.
2. World Health Organization. International statistical classification of diseases and related health problems (ICD-10): World Health Organization; 1994.
3. Head J, Ferrie JE, Alexanderson K, et al. Diagnosis-specific sickness absence as a predictor of mortality: the Whitehall II prospective cohort study. *BMJ* 2008; **337**: a1469.

Description of covariates

The covariates included age, sex, socioeconomic status (SES), and chronic disease. SES was based on occupational class, except in HeSSup, in wich information on occupational class was unavailable and SES was based on vocational educational attainment. In FPS and GAZEL, SES was based on register data, and in HeSSup and Whitehall II, it was based on self-reports. High SES included administrators, managers, experts, and specialists; and in HeSSup, those with a university/polytechnic degree. Intermediate SES included skilled non-manual occupations, such as higher executive officers (Whitehall II), office workers, customer service workers, sales workers, and hospital nurses; and in HeSSup, those with a college-level education. Low SES included manual workers, such as those in construction, manufacturing, and transportation; in HeSSup, those with vocational school, vocational course, and apprenticeship training or no vocational education; and in Whitehall II, low SES included clerical and office support workers.

In FPS and HeSSup, information on chronic disease was derived from health registers and a check-list administered in the survey, and included coronary heart disease, stroke, diabetes, lung disease, asthma, cancer, musculoskeletal disease, and mental disorders. In GAZEL, it was derived from survey questions on asthma, hypertension, angina, myocardial infarction, stroke, rheumatoid arthritis, diabetes, and cancer. Whitehall II used survey responses regarding the presence of any longstanding illness (yes/no) and the type of disease. Chronic disease was defined as coronary heart disease, stroke, respiratory disease, asthma, musculoskeletal disease, cancer, depression, or other mental disorder.

Description of Population Attributable Fraction (PAF)

We used the following formula to calculate PAF:

$$PAF\ in\ group\ j = P_j(\ RR_j - 1)/[(\sum_{i=1}^{K} P_i(\ RR_i - 1)) + 1],$$

where P_i = proportion of the population in group i; RR_i = rate ratio in group i; K = number of non-reference risk groups

Reference: Hanley JA. A heuristic approach to the formulas for population attributable fraction. J Epidemiol Community Health 2001;55:508-14.

PAFs were calculated for both the current data and the external source-derived prevalence levels of the risk factors. We estimated 95% confidence intervals for PAF using 95% confidence intervals for summary rate ratios across all four cohort studies.

Data sources for external prevalences:

Smoking, heavy episodic drinking, overweight and obesity:
EUROSTAT (https://ec.europa.eu/eurostat, accessed Sept 5, 2018)

Alcohol consumption:
Wood AM, Kaptoge S, Butterworth AS, et al. Risk thresholds for alcohol consumption: combined analysis of individual-participant data for 599 912 current drinkers in 83 prospective studies. Lancet 2018; 391: 1513–23.

Low physical activity:
Guthold R, Stevens GA, Riley LM, Bull FC. Worldwide trends in insufficient physical activity from 2001 to 2016: a pooled analysis of 358 population-based surveys with 1·9 million participants. Lancet Glob Health 2018; Published Online September 4, 2018 http://dx.doi.org/10.1016/ S2214-109X(18)30357-7.

Supplementary Table 1: Rate ratio (RR) and 95% confidence intervals (CI) for association between lifestyle factors and sickness absence due to musculoskeletal diseases in each study cohort

Lifestyle factor and study cohort	RR*	(95% CI)*
Smoking		
FPS	1.38	(1.24-1.53)
HESSUP	1.46	(1.13-1.88)
GAZEL	0.82	(0.64-1.05)
Whitehall II	2.65	(1.84-3.83)
High alcohol consumption		
FPS	0.99	(0.88-1.11)
HESSUP	0.89	(0.69-1.14)
GAZEL	1.07	(0.86-1.33)
Whitehall II	1.88	(1.36-2.59)
Heavy episodic drinking		
FPS	0.99	(0.85-1.16)
HESSUP	1.09	(0.80-1.50)
Overweight		
FPS	1.29	(1.18-1.41)
HESSUP	1.37	(1.10-1.71)
GAZEL	1.18	(0.95-1.46)
Whitehall II	1.55	(1.16-2.06)
Obesity		
FPS	1.63	(1.44-1.85)
HESSUP	1.84	(1.33-2.55)
GAZEL	1.57	(1.10-2.25)
Whitehall II	0.86	(0.55-1.37)
Low physical activity		
FPS	1.23	(1.11-1.36)
HESSUP	1.06	(0.83-1.36)
GAZEL	1.34	(1.09-1.64)
Whitehall II	1.37	(0.97-1.91)

*Adjusted for age, sex, socioeconomic status and chronic disease.

Supplementary Table 2: Rate ratio (RR) and 95% confidence intervals (CI) for association between lifestyle factors and sickness absence due to depressive disorders in each study cohort

Lifestyle factor and study cohort	RR*	(95 % CI)*
Smoking		
FPS	1.56	(1.24-1.96)
HESSUP	1.36	(0.83-2.23)
GAZEL	2.52	(1.51-4.23)
Whitehall II	2.10	(1.34-3.29)
High alcohol consumption		
FPS	1.20	(0.95-1.51)
HESSUP	0.85	(0.51-1.41)
GAZEL	1.07	(0.68-1.68)
Whitehall II	2.30	(1.53-3.46)
Heavy episodic drinking		
FPS	1.82	(1.30-2.54)
HESSUP	2.22	(1.15-4.29)
Overweight		
FPS	1.01	(0.84-1.23)
HESSUP	0.87	(0.56-1.37)
GAZEL	1.79	(1.16-2.75)
Whitehall II	1.01	(0.71-1.44)
Obesity		
FPS	1.20	(0.92-1.56)
HESSUP	1.55	(0.81-2.96)
GAZEL	2.32	(1.12-4.81)
Whitehall II	1.80	(1.01-3.22)
Low physical activity		
FPS	1.71	(1.38-2.11)
HESSUP	1.47	(0.90-2.41)
GAZEL	1.73	(1.12-2.66)
Whitehall II	1.61	(1.07-2.40)

*Adjusted for age, sex, socioeconomic status and chronic disease.

Supplementary Table 3: Rate ratio (RR) and 95% confidence intervals (CI) for association between lifestyle factors and sickness absence due to external causes in each study cohort

Lifestyle factor and study cohort	RR*	(95% CI)*
Smoking		
FPS	1.45	(1.23-1.71)
HESSUP	1.27	(0.90-1.79)
GAZEL	1.43	(1.08-1.89)
Whitehall II	0.92	(0.63-1.34)
High alcohol consumption		
FPS	1.37	(1.15-1.63)
HESSUP	1.21	(0.86-1.71)
GAZEL	1.25	(0.98-1.59)
Whitehall II	0.75	(0.55-1.03)
Heavy episodic drinking		
FPS	1.61	(1.26-2.06)
HESSUP	1.72	(1.13-2.63)
Overweight		
FPS	1.33	(1.16-1.53)
HESSUP	1.02	(0.75-1.39)
GAZEL	1.06	(0.83-1.34)
Whitehall II	1.06	(0.79-1.42)
Obesity		
FPS	1.49	(1.23-1.81)
HESSUP	1.27	(0.82-1.98)
GAZEL	1.12	(0.75-1.66)
Whitehall II	2.42	(1.53-3.83)
Low physical activity		
FPS	0.95	(0.82-1.11)
HESSUP	0.89	(0.63-1.26)
GAZEL	0.81	(0.64-1.02)
Whitehall II	1.24	(0.88-1.74)

*Adjusted for age, sex, socioeconomic status and chronic disease.

Supplementary Table 4: Rate ratio (RR) and 95% confidence intervals (CI) for association between lifestyle factors and sickness absence due to circulatory diseases in each study cohort

Lifestyle factor and study cohort	RR*	(95% CI)*
Smoking		
FPS	1.61	(1.24-2.08)
HESSUP	1.27	(0.69-2.31)
GAZEL	1.92	(1.18-3.15)
Whitehall II	1.27	(0.37-4.30)
High alcohol consumption		
FPS	0.73	(0.55-0.96)
HESSUP	0.98	(0.52-1.85)
GAZEL	1.27	(0.83-1.93)
Whitehall II	0.77	(0.28-2.09)
Heavy episodic drinking		
FPS	1.13	(0.77-1.67)
HESSUP	1.55	(0.74-3.25)
Overweight		
FPS	1.34	(1.07-1.68)
HESSUP	1.17	(0.69-1.97)
GAZEL	0.76	(0.51-1.15)
Whitehall II	0.97	(0.39-2.42)
Obesity		
FPS	1.69	(1.24-2.30)
HESSUP	1.83	(0.87-3.85)
GAZEL	2.50	(1.27-4.93)
Whitehall II	2.15	(0.47-9.89)
Low physical activity		
FPS	1.09	(0.85-1.41)
HESSUP	1.22	(0.67-2.21)
GAZEL	2.08	(1.39-3.10)
Whitehall II	0.37	(0.12-1.10)

*Adjusted for age, sex, socioeconomic status and chronic disease.

Supplementary Table 5: Rate ratio (RR) and 95% confidence intervals (CI) for association between lifestyle factors and sickness absence due to respiratory diseases in each study cohort

Lifestyle factor and study cohort	RR*	(95% CI)*
Smoking		
FPS	1.50	(1.21-1.86)
HESSUP	1.29	(0.76-2.19)
GAZEL	1.27	(1.05-1.53)
Whitehall II	1.19	(1.03-1.36)
High alcohol consumption		
FPS	1.10	(0.89-1.37)
HESSUP	0.88	(0.52-1.49)
GAZEL	1.01	(0.85-1.19)
Whitehall II	1.15	(1.02-1.29)
Heavy episodic drinking		
FPS	1.35	(0.99-1.84)
HESSUP	1.27	(0.66-2.45)
Overweight		
FPS	1.12	(0.94-1.34)
HESSUP	0.96	(0.59-1.56)
GAZEL	0.93	(0.79-1.09)
Whitehall II	1.02	(0.92-1.14)
Obesity		
FPS	1.76	(1.37-2.25)
HESSUP	2.26	(1.20-4.25)
GAZEL	1.12	(0.86-1.46)
Whitehall II	1.36	(1.14-1.62)
Low physical activity		
FPS	1.50	(1.22-1.84)
HESSUP	1.75	(1.06-2.89)
GAZEL	1.40	(1.19-1.64)
Whitehall II	1.28	(1.13-1.46)

*Adjusted for age, sex, socioeconomic status and chronic disease.

Supplementary Table 6: Rate ratio (RR) and 95% confidence intervals (CI) for association between lifestyle factors and sickness absence due to digestive diseases in each study cohort

Lifestyle factor and study cohort	RR*	(95% CI)*
Smoking		
FPS	1.21	(0.92-1.60)
HESSUP	0.96	(0.56-1.64)
GAZEL	0.83	(0.61-1.13)
Whitehall II	1.47	(1.00-2.17)
High alcohol consumption		
FPS	0.91	(0.69-1.20)
HESSUP	0.91	(0.54-1.53)
GAZEL	1.30	(0.98-1.71)
Whitehall II	1.13	(0.80-1.59)
Heavy episodic drinking		
FPS	0.79	(0.53-1.17)
HESSUP	0.94	(0.49-1.83)
Overweight		
FPS	0.98	(0.78-1.23)
HESSUP	1.06	(0.65-1.71)
GAZEL	1.22	(0.93-1.61)
Whitehall II	0.70	(0.52-0.95)
Obesity		
FPS	1.63	(1.19-2.23)
HESSUP	1.40	(0.71-2.77)
GAZEL	1.85	(1.17-2.92)
Whitehall II	1.71	(1.04-2.82)
Low physical activity		
FPS	1.35	(1.05-1.74)
HESSUP	0.71	(0.42-1.19)
GAZEL	1.48	(1.15-1.90)
Whitehall II	1.04	(0.75-1.46)

*Adjusted for age, sex, socioeconomic status and chronic disease.

Supplementary Figure 1: Rate ratio (RR) and 95% confidence intervals (CI) from meta-analyses for association between lifestyle factors and diagnosis-specific sickness absence in the subgroups of sick-listed participants, adjusted for age, sex, socioeconomic status and chronic disease. Outcomes are sickness absence at follow-up due to the same diagnosis (longer duration or repeated absence)

Diagnosis of sickness absence (average absence rate due to the same diagnosis)	Lifestyle factor	Rate ratio (95% confidence interval)	N of cases with absence	I^2	p-value
Musculoskeletal diseases (rate 278.4 days per 10 person-years)	Obesity	1.19 (1.13—1.25)	17 218	29.9	0.23
	Low physical activity	1.17 (1.12—1.22)	17 558	28.1	0.24
	Smoking	1.10 (1.05—1.15)	17 354	92.4	0.000
	Overweight	1.08 (1.04—1.12)	17 218	71.5	0.015
	High alcohol consumption	0.98 (0.93—1.03)	17 692	83.6	0.000
	Heavy episodic drinking	0.95 (0.88—1.02)	15 076	0.0	0.33
Depressive disorders (rate 327.1 days per 10 person-years)	Low physical activity	1.24 (1.14—1.34)	5585	46.9	0.13
	Smoking	1.09 (1.00—1.19)	5530	10.0	0.34
	Heavy episodic drinking	1.06 (0.93—1.21)	4356	74.3	0.049
	Obesity	1.06 (0.95—1.17)	5493	0.0	0.85
	High alcohol consumption	0.99 (0.90—1.08)	5644	90.9	0.000
	Overweight	0.99 (0.91—1.08)	5493	63.1	0.043
External causes (rate 98.9 days per 10 person-years)	Obesity	1.20 (1.13—1.28)	8882	64.7	0.037
	Heavy episodic drinking	1.13 (1.05—1.21)	6788	0.0	0.97
	Smoking	1.07 (1.02—1.13)	8920	77.3	0.004
	Overweight	1.07 (1.02—1.12)	8882	64.0	0.040
	High alcohol consumption	1.02 (0.96—1.08)	9077	45.8	0.14
	Low physical activity	0.98 (0.93—1.04)	8982	48.1	0.12
Circulatory diseases (rate 193.6 per 10 person-years)	Smoking	1.50 (1.35—1.68)	3576	44.4	0.15
	Heavy episodic drinking	1.30 (1.08—1.56)	2930	28.4	0.24
	Low physical activity	1.16 (1.05—1.28)	3608	88.7	0.000
	Obesity	1.15 (1.02—1.29)	3567	28.4	0.24
	Overweight	1.00 (0.90—1.10)	3567	28.9	0.24
	High alcohol consumption	0.97 (0.86—1.09)	3634	81.2	0.001
Respiratory diseases (rate 48.4 days per 10 person-years)	Low physical activity	1.30 (1.24—1.37)	8456	31.4	0.22
	Obesity	1.18 (1.10—1.26)	8395	47.5	0.13
	Heavy episodic drinking	1.17 (1.03—1.32)	3723	0.0	0.66
	Smoking	1.12 (1.06—1.19)	8491	23.0	0.27
	High alcohol consumption	1.12 (1.06—1.18)	8597	68.0	0.025
	Overweight	0.93 (0.88—0.97)	8395	51.8	0.10
Digestive diseases (rate 54.0 days per 10 person-years)	Smoking	1.10 (1.02—1.20)	4148	0.0	0.45
	Obesity	1.07 (0.98—1.17)	4084	87.3	0.000
	Low physical activity	1.05 (0.97—1.12)	4121	84.8	0.000
	High alcohol consumption	1.03 (0.95—1.11)	4185	81.7	0.001
	Heavy episodic drinking	0.89 (0.79—1.01)	2482	83.7	0.013
	Overweight	0.84 (0.79—0.90)	4084	72.4	0.012

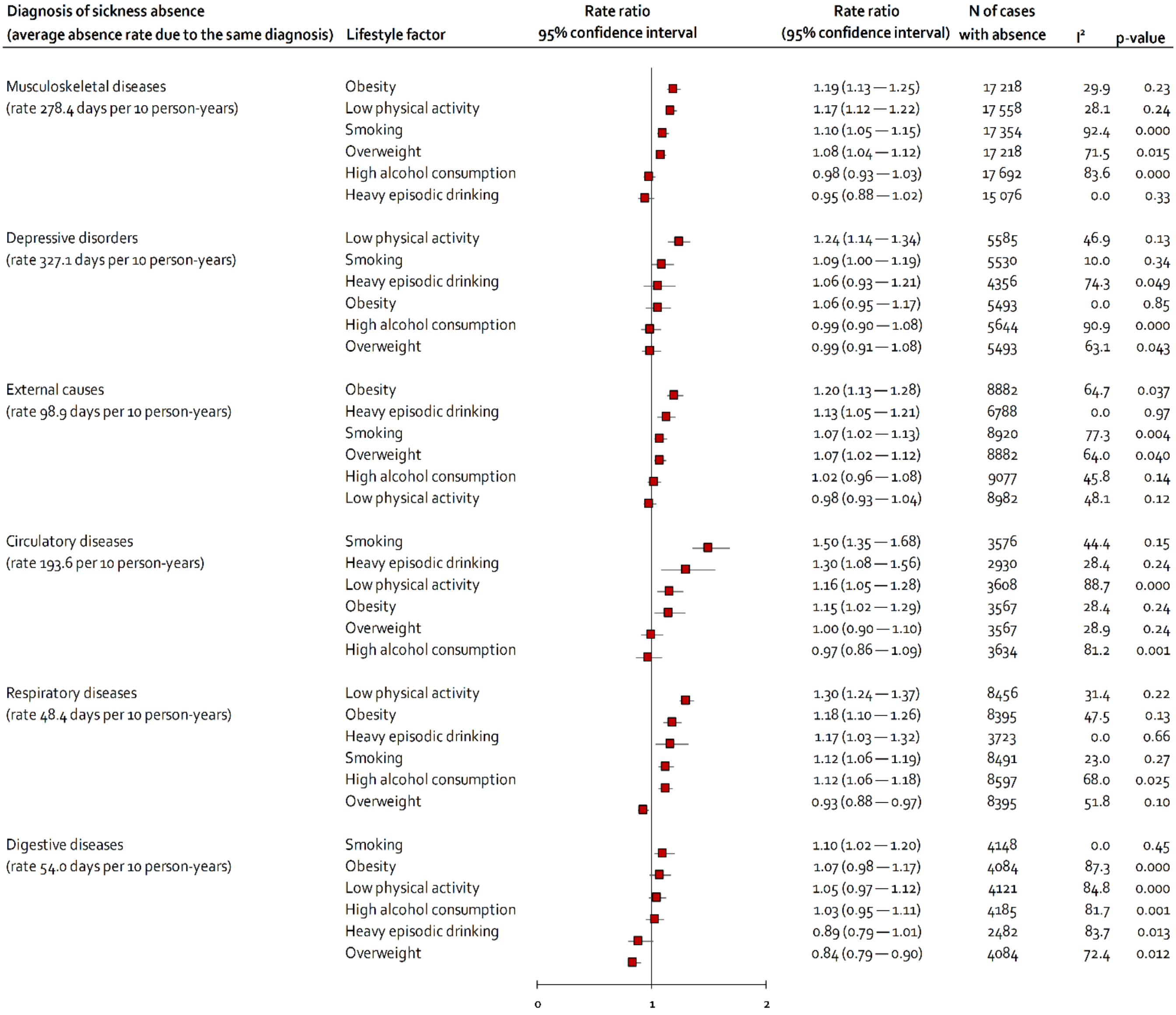

Supplementary Figure 2: Rate ratio (RR) and 95% confidence intervals (CI) from meta-analyses for association between lifestyle factors and diagnosis-specific sickness absence in the subgroups of sick-listed participants, adjusted for age, sex, socioeconomic status and chronic disease. Outcomes are sickness absence at follow-up due to other diagnoses (multiple-causes absence)

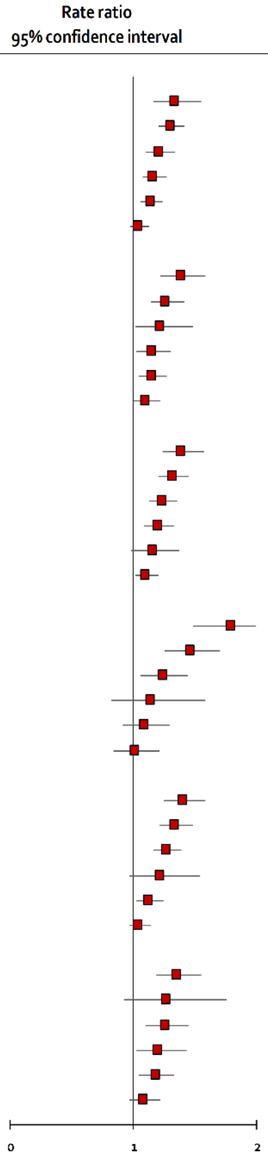

Diagnosis of sickness absence (average absence rate due to other diagnoses)	Lifestyle factor	Rate ratio (95% confidence interval)	N of cases with absence	I^2	p-value
Musculoskeletal diseases (rate 126.7 days per 10 person-years)	Heavy episodic drinking	1.34 (1.16 — 1.55)	15 076	68.3	0.08
	Smoking	1.30 (1.20 — 1.41)	17 354	82.9	0.001
	Obesity	1.21 (1.10 — 1.34)	17 218	0.0	0.49
	High alcohol consumption	1.16 (1.07 — 1.27)	17 692	0.0	0.43
	Low physical activity	1.14 (1.06 — 1.23)	17 558	35.9	0.20
	Overweight	1.04 (0.97 — 1.12)	17 218	48.5	0.12
Depressive disorders (rate 190.6 days per 10 person-years)	Obesity	1.39 (1.22 — 1.58)	5493	47.1	0.13
	Smoking	1.26 (1.14 — 1.41)	5530	0.0	0.76
	Heavy episodic drinking	1.22 (1.01 — 1.48)	4356	0.0	0.69
	High alcohol consumption	1.15 (1.02 — 1.30)	5644	0.4	0.39
	Overweight	1.15 (1.04 — 1.27)	5493	49.9	0.11
	Low physical activity	1.10 (1.00 — 1.22)	5585	0.0	0.77
External causes (rate 171.2 days per 10 person-years)	Obesity	1.39 (1.23 — 1.57)	8882	37.6	0.19
	Smoking	1.32 (1.20 — 1.45)	8920	0.0	0.75
	Low physical activity	1.23 (1.12 — 1.35)	8982	0.0	0.88
	High alcohol consumption	1.20 (1.08 — 1.33)	9077	77.4	0.004
	Heavy episodic drinking	1.16 (0.98 — 1.37)	6788	0.0	0.40
	Overweight	1.10 (1.01 — 1.20)	8882	66.3	0.031
Circulatory diseases (rate 190.6 days per 10 person-years)	Obesity	1.79 (1.48 — 2.16)	3567	0.0	0.59
	Low physical activity	1.46 (1.25 — 1.70)	3608	46.3	0.13
	Overweight	1.24 (1.06 — 1.44)	3567	0.0	0.60
	Heavy episodic drinking	1.14 (0.82 — 1.58)	2930	0.0	0.47
	Smoking	1.09 (0.91 — 1.29)	3576	32.1	0.22
	High alcohol consumption	1.01 (0.84 — 1.21)	3634	41.4	0.16
Respiratory diseases (rate 177.9 days per 10 person-years)	Obesity	1.40 (1.24 — 1.58)	8395	29.7	0.23
	Smoking	1.34 (1.21 — 1.48)	8491	0.0	0.50
	Low physical activity	1.27 (1.16 — 1.39)	8456	71.5	0.015
	Heavy episodic drinking	1.22 (0.96 — 1.54)	3723	0.0	1.00
	High alcohol consumption	1.12 (1.02 — 1.24)	8597	0.0	0.67
	Overweight	1.04 (0.96 — 1.14)	8395	66.6	0.030
Digestive diseases (rate 162.8 days per 10 person-years)	Smoking	1.35 (1.18 — 1.55)	4148	50.0	0.11
	Heavy episodic drinking	1.27 (0.92 — 1.75)	2482	0.0	0.77
	High alcohol consumption	1.26 (1.10 — 1.45)	4185	75.4	0.007
	Obesity	1.20 (1.02 — 1.43)	4084	58.9	0.06
	Low physical activity	1.18 (1.04 — 1.33)	4121	29.3	0.24
	Overweight	1.08 (0.96 — 1.22)	4084	59.7	0.06

Supplementary Table 7: Summary estimates from meta-analyses for association between lifestyle factors and sickness absence days due to specific diagnoses in the total population of three cohort studies (FPS, HeSSup, Gazel), excluding the Whitehall II cohort

	Depressive disorders	Circulatory diseases	Musculoskeletal diseases	Digestive diseases	Respiratory diseases	External causes
Lifestyle factors	Rate ratio (95% CI)*	Rate ratio (95% CI)*	Rate ratio (95% CI)*	Rate ratio (95% CI)*	Rate ratio (95% CI)*	Rate ratio (95% CI)*
Smoking (n=68 166)						
Non-smoking	1.00	1.00	1.00	1.00	1.00	1.00
Smoking	1.63 (1.35-1.98)	1.62 (1.30-2.00)	1.30 (1.18-1.42)	1.01 (0.84-1.23)	1.36 (1.19-1.56)	1.42 (1.24-1.62)
I^2 (p-value)	41.3% (0.18)	0.0% (0.58)	87.0% (0.000)	38.0% (0.20)	0.0% (0.51)	0.0% (0.79)
Alcohol consumption (n=69 141)						
Moderate	1.00	1.00	1.00	1.00	1.00	1.00
High consumption	1.12 (0.93-1.36)	0.88 (0.70-1.09)	0.99 (0.90-1.09)	1.06 (0.88-1.28)	1.03 (0.91-1.17)	1.31 (1.15-1.49)
I^2 (p-value)	0.0% (0.47)	57.8% (0.09)	0.0% (0.56)	43.9 (0.17)	0.0% (0.69)	0.0% (0.74)
Body mass index (n=67 622)						
Normal weight	1.00	1.00	1.00	1.00	1.00	1.00
Overweight	1.07 (0.91-1.26)	1.17 (0.97-1.41)	1.28 (1.19-1.39)	1.07 (0.91-1.26)	1.01 (0.90-1.13)	1.22 (1.09-1.37)
Obesity	1.33 (1.05-1.67)	1.81 (1.39-2.35)	1.65 (1.47-1.84)	1.66 (1.30-2.11)	1.48 (1.24-1.76)	1.39 (1.18-1.64)
I^2 (p-value)	Overweight: 69.8% (0.036);<br>Obesity: 34.1% (0.22)	Overweight: 65.0% (0.06)<br>Obesity: 0.0% (0.59)	Overweight: 0.0% (0.63)<br>Obesity: 0.0% (0.76)	Overweight: 0.0% (0.48)<br>Obesity: 0.0% (0.79)	Overweight: 15.2% (0.31)<br>Obesity: 74.5 (0.020)	Overweight: 51.3% (0.13)<br>Obesity: 0.0% (0.41)
Physical activity (n=68 544)						
Physically active	1.00	1.00	1.00	1.00	1.00	1.00
Low activity	1.68 (1.41-2.01)	1.30 (1.06-1.59)	1.23 (1.13-1.34)	1.32 (1.11-1.56)	1.45 (1.29-1.64)	0.90 (0.80-1.02)
I^2 (p-value)	0.0% (0.85)	72.2% (0.028)	3.2% (0.36)	68.1% (0.043)	0.0% (0.66)	0.0% (0.53)

*Adjusted for age, sex, socioeconomic status and chronic disease.

Supplementary Table 8: Summary estimates from meta-analyses for association between lifestyle factors and sickness absence days due to specific diagnoses in the total population of three cohort studies (FPS, HeSSup, Whitehall II), excluding the GAZEL cohort

	Depressive disorders	Circulatory diseases	Musculoskeletal diseases	Digestive diseases	Respiratory diseases	External causes
Lifestyle factors	Rate ratio (95% CI)*	Rate ratio (95% CI)*	Rate ratio (95% CI)*	Rate ratio (95% CI)*	Rate ratio (95% CI)*	Rate ratio (95% CI)*
Smoking (n=62 129)						
Non-smoking	1.00	1.00	1.00	1.00	1.00	1.00
Smoking	1.61 (1.33-1.95)	1.54 (1.22-1.95)	1.45 (1.32-1.59)	1.24 (1.00-1.52)	1.27 (1.14-1.43)	1.34 (1.16-1.53)
I^2 (p-value)	0.0% (0.39)	0.0% (0.74)	82.2% (0.004)	0.0% (0.44)	36.4% (0.21)	58.2% (0.09)
Alcohol consumption (n=63 316)						
Moderate	1.00	1.00	1.00	1.00	1.00	1.00
High consumption	1.31 (1.09-1.58)	0.77 (0.60-0.98)	1.04 (0.94-1.14)	0.98 (0.80-1.19)	1.13 (1.02-1.25)	1.19 (1.04-1.37)
I^2 (p-value)	81.2% (0.005)	0.0% (0.71)	86.8% (0.001)	0.0% (0.60)	0.0% (0.60)	81.5% (0.004)
Body mass index (n=61 542)						
Normal weight	1.00	1.00	1.00	1.00	1.00	1.00
Overweight	0.99 (0.85-1.16)	1.29 (1.06-1.58)	1.32 (1.22-1.43)	0.89 (0.75-1.05)	1.04 (0.95-1.14)	1.24 (1.10-1.39)
Obesity	1.32 (1.05-1.65)	1.72 (1.30-2.28)	1.59 (1.42-1.78)	1.62 (1.26-2.07)	1.51 (1.32-1.74)	1.55 (1.32-1.83)
I^2 (p-value)	Overweight: 0.0% (0.83)	Overweight: 0.0% (0.74)	Overweight: 0.0% (0.46)	Overweight: 44.9% (0.16)	Overweight: 0.0% (0.64)	Overweight: 44.8% (0.16)
	Obesity: 0.0% (0.40)	Obesity: 0.0% (0.94)	Obesity: 74.7% (0.019)	Obesity: 0.0% (0.90)	Obesity: 54.4% (0.11)	Obesity: 56.2% (0.10)
Physical activity (n=62 204)						
Physically active	1.00	1.00	1.00	1.00	1.00	1.00
Low activity	1.66 (1.39-1.98)	1.06 (0.84-1.33)	1.21 (1.11-1.33)	1.14 (0.95-1.38)	1.35 (1.22-1.51)	0.98 (0.86-1.11)
I^2 (p-value)	0.0% (0.85)	46.3% (0.15)	0.0% (0.43)	61.5% (0.08)	25.9% (0.26)	12.6% (0.32)

*Adjusted for age, sex, socioeconomic status and chronic disease.
